# Influence of the Accumulation of Unhealthy Eating Habits on Obesity in a General Japanese Population: The Hisayama Study

**DOI:** 10.3390/nu12103160

**Published:** 2020-10-16

**Authors:** Yuki Ishida, Daigo Yoshida, Takanori Honda, Yoichiro Hirakawa, Mao Shibata, Satoko Sakata, Yoshihiko Furuta, Emi Oishi, Jun Hata, Takanari Kitazono, Toshiharu Ninomiya

**Affiliations:** 1 Department of Epidemiology and Public Health, Graduate School of Medical Sciences, Kyushu University, Fukuoka 812-8582, Japan; ishida-y@eph.med.kyushu-u.ac.jp (Y.I.); honda-t@eph.med.kyushu-u.ac.jp (T.H.); you1@eph.med.kyushu-u.ac.jp (Y.H.); shibata.mao.276@m.kyushu-u.ac.jp (M.S.); ssakata@eph.med.kyushu-u.ac.jp (S.S.); furuta.yoshihiko.496@m.kyushu-u.ac.jp (Y.F.); oishiemi@eph.med.kyushu-u.ac.jp (E.O.); junhata@eph.med.kyushu-u.ac.jp (J.H.); nino@eph.med.kyushu-u.ac.jp (T.N.); 2 Department of Medicine and Clinical Science, Graduate School of Medical Sciences, Kyushu University, Fukuoka 812-8582, Japan; kitazono@intmed2.med.kyushu-u.ac.jp; 3 Center for Cohort Studies, Graduate School of Medical Sciences, Kyushu University, Fukuoka 812-8582, Japan

**Keywords:** unhealthy eating habits, accumulation, obesity, central obesity, general Japanese population

## Abstract

Few studies have examined the association between the accumulation of unhealthy eating habits and the likelihood of obesity or central obesity in a general Japanese population. We examined this association in a sample of 1906 community-dwelling Japanese subjects (age: 40–74 years) who participated in a health check-up in 2014. A face-to-face questionnaire interview was conducted to collect information about three unhealthy eating habits, i.e., snacking, eating quickly, and eating late-evening meals. Obesity was defined as body mass index ≥25 kg/m^2^ and central obesity was defined as waist circumference ≥90 cm in men and ≥80 cm in women. The odds ratios (OR) were estimated by using a logistic regression analysis. Subjects with any one of the three eating habits had a significantly higher likelihood of obesity than those without that habit after adjusting for confounding factors. The multivariable-adjusted OR for obesity increased linearly with an increase in the number of accumulated unhealthy eating habits (*p* for trend < 0.001). Similar associations were observed for central obesity. Our findings suggest that modifying each unhealthy eating habit and avoiding an accumulation of multiple unhealthy eating habits might be important to reduce the likelihood of obesity.

## 1. Introduction

The number of people with obesity is increasing globally [1]. Obesity is a major risk factor for chronic diseases, such as hypertension, diabetes, hyperlipidemia, cardiovascular disease, and cancer [2]. Central obesity, defined by an increased waist circumference, has also been reported to increase the risk of cardiovascular disease and death [3,4]. In order to reduce the burden of obesity-related diseases, the prevention of obesity must be a public health priority.

Among the various strategies for health promotion, one commonality is the importance of healthy eating habits to prevent obesity [2]. Several epidemiologic studies have indicated that unhealthy eating habits, such as snacking [5,6,7,8], eating quickly [7,9,10], and eating late-evening meals [11,12], are significantly associated with an increased risk of obesity or central obesity. These previous studies investigated the influence of each eating habit separately, but it is also important to consider the influence of the accumulation of unhealthy eating habits on obesity and central obesity, since unhealthy eating habits tend to overlap. However, there have been few population-based studies evaluating the influence of the accumulation of multiple unhealthy eating habits on having obesity and central obesity in Japanese.

Therefore, the aim of the present study was to examine the associations of both individual and accumulated unhealthy eating habits with the likelihood of having obesity and central obesity in a general Japanese population.

## 2. Materials and Methods

### 2.1. Study Population

The Hisayama Study is a population-based prospective cohort study of cardiovascular disease and its risk factors, which was begun in 1961 in the town of Hisayama, a suburb of the Fukuoka metropolitan area on Kyushu Island, Japan. According to the national census, the age and occupational distributions in Hisayama have been almost identical to those of all of Japan since the 1960s [13,14]. The present cross-sectional study was based on a screening survey conducted in 2014. A total of 1930 residents aged 40–74 years (51.7% of the total population of this age group) underwent a health check-up and completed an interviewer-administered questionnaire about eating habits. After excluding 4 individuals who did not provide consent to participate in the study and 20 without available data of eating habits, the remaining 1906 subjects (835 men and 1071 women) were enrolled in this study.

### 2.2. Definition of Obesity and Central Obesity

Body height and weight were measured using an automated digital scale (DC-250, Tanita, Tokyo, Japan) in light clothing without shoes, and body mass index (BMI) was calculated as weight (kg) divided by height squared (m^2^). Obesity was defined as a BMI ≥25 kg/m^2^. Waist circumference at the umbilical level was measured by trained nurses using a non-stretchable tape measure with the participant in the standing position, and central obesity was defined as a waist circumference ≥90 cm in men and ≥80 cm in women according to International Obesity Task Force central obesity criteria for Asia [15].

### 2.3. Definition of Unhealthy Eating Habits

A face-to-face interview by registered dietitians was conducted to collect the information on eating habits using a questionnaire, which was modified from the questionnaire for the Standard Health Check-up and Counseling Guidance to prevent metabolic syndrome proposed by the Japanese Ministry of Health, Labour and Welfare [16]. The original questionnaire is widely used in the nationwide health check-ups for residents aged 40 to 74 years in Japan.

Eating habits were determined by the following questions: “Do you eat snacks?” (snacking); “Does your eating speed more quickly than other people?” (eating quickly); “Do you have late-evening meals within two hours before bedtime?” (eating late-evening meals). The answer options were “yes” or “no”. Those who answered yes to a question were defined as having that particular unhealthy eating habit. The number of accumulated unhealthy eating habits was determined by summing up the positive responses, ranging from 0 to 3.

### 2.4. Measurement of Other Risk Factors

Each participant completed a self-administered questionnaire including smoking habits, drinking habits, regular exercise, marital status, living status, and employment status. Smoking habits and drinking habits were classified into currently habitual or not. The subjects who reported engaging in sports or other forms of exertion ≥3 times a week during their leisure time made up the regular exercise group. Marital status was classified as currently “married” or “unmarried, divorced, or widowed”. Living status was categorized as “living alone” or “living with others”. Employment status was categorized as currently “employed” or “unemployed”; housewives were classified as “unemployed” in the present study. The questionnaire was checked by trained interviewers at the screening.

### 2.5. Statistical Analysis

Descriptive statistics according to the response to each unhealthy eating habit and the number of accumulated eating habits were presented as age- and sex-adjusted means or frequencies. The group differences were tested by analysis of covariance and a logistic regression model. The age- and sex-adjusted mean values of BMI and waist circumference according to the status of each unhealthy eating habit and the numbers of accumulated eating habits were estimated by using the analysis of covariance. The means of BMI and waist circumference across the numbers of accumulated unhealthy eating habits were tested by a linear regression model. The odds ratios (OR) and their 95% confidence intervals (CIs) for the presence of obesity and central obesity according to each unhealthy eating habit and the number of accumulated eating habits were computed with the use of the logistic regression model. The trends in the estimates across the number of accumulated unhealthy eating habits were tested by including the ordinal number (0, 1, 2, or 3) representing the number of the accumulated eating habits in the relevant model. The heterogeneities in the association between subgroup covariates were tested by adding the multiplicative interaction term to the relevant model. All statistical analyses were performed using the SAS program package version 9.4 (SAS Institute Inc., Cary, NC, USA). Two-tailed *p*-values of < 0.05 were considered significant.

### 2.6. Ethical Considerations

The study protocol was approved by the Kyushu University Institutional Review Board for Clinical Research, and the procedures followed were in accordance with national guidelines. All participants provided written informed consent.

## 3. Results

Table 1 shows the age- and sex-adjusted mean values or frequencies of covariates according to the status of each of the unhealthy eating habits. Subjects who snacked were more likely to be women, and less likely to be current smokers, current drinkers, living alone, and employed than those without. Subjects who ate quickly were younger than those who were not. Subjects who ate late-evening meals were younger, and more likely to be men, current smokers, current drinkers, living alone, and employed than those who did not.

Among the 1906 subjects, 504 (26.4%) had obesity and 860 (45.1%) had central obesity. As shown in Table 2, the age- and sex-adjusted mean values of BMI and waist circumference were higher in the subjects with any one of the unhealthy eating habits than in those without that habit (all *p* values < 0.05; except for BMI in subjects who ate late-evening meals). Subjects with any one of the unhealthy eating habits had a significantly greater likelihood of the presence of obesity (snacking: OR 1.49 [95% CI 1.19–1.86]; eating quickly: 2.11 [1.71–2.61]; eating late-evening meals: 1.39 [1.09–1.77]) and central obesity (snacking: 1.29 [1.05–1.58]; eating quickly: 1.89 [1.55–2.30]; eating late-evening meals: 1.36 [1.08–1.72]) after adjusting for age, sex, current smoking, current drinking, regular exercise, marital status, living status, and employment status.

**Figure 1:**
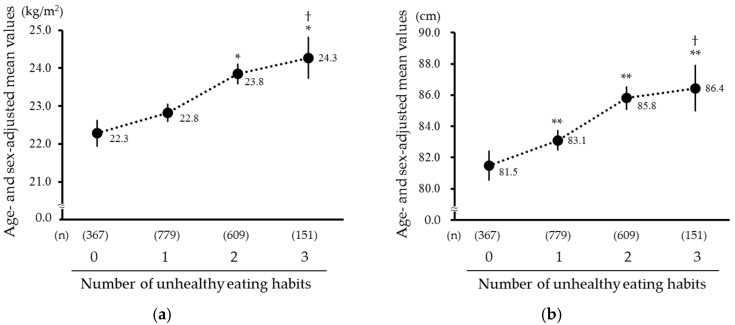
Age- and sex-adjusted mean values of body mass index and waist circumference according to the number of accumulated unhealthy eating habits: (**a**) Body mass index; (**b**) Waist circumference. Solid circles and vertical bars represent the mean values and 95% confidence intervals of each parameter, respectively. * *p* < 0.05, ** *p* < 0.01 vs. “0”, † *p* for trend < 0.001.

**Figure 2:**
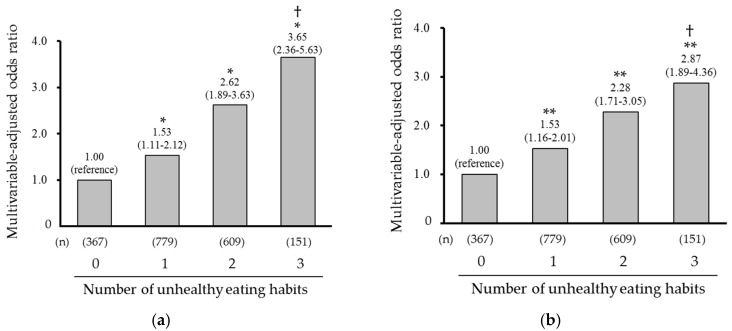
Multivariable-adjusted odds ratio of obesity and central obesity according to the number of accumulated unhealthy eating habits: (**a**) Obesity; (**b**) Central obesity. The values on the bars show the odds ratios (95% confidence intervals), which were adjusted for age, sex, current smoking, current drinking, regular exercise, marital status, living status, and employment status. * *p* < 0.05, ** *p* < 0.01 vs. “0”, † *p* for trend < 0.001.

Next, we investigated the association between the number of accumulated unhealthy eating habits and the likelihood of obesity and central obesity. Descriptive statistics according to the number of accumulated unhealthy eating habits are shown in Table 3. Subjects with a higher number of accumulated unhealthy eating habits were more likely to be younger. A higher number of accumulated unhealthy eating habits were significantly associated with the age- and sex-adjusted mean values of BMI and waist circumference (both *p* for trend < 0.001; Figure 1). The multivariable-adjusted OR for having obesity or central obesity increased linearly with a higher number of accumulated unhealthy eating habits (obesity: OR 1.53 [95% CI 1.11–2.12], 2.62 [1.89–3.63], and 3.65 [2.36–5.63] for one, two, and three unhealthy eating habits, respectively, *p* for trend < 0.001; central obesity: 1.53 [1.16–2.01], 2.28 [1.71–3.05], and 2.87 [1.89–4.36], *p* for trend < 0.001; Figure 2).

Finally, we compared the age- and sex-adjusted ORs of the presence of obesity and central obesity per every one increment in the number of accumulated unhealthy eating habits between the subgroups of covariates (Table 4). The magnitudes of the association between the number of accumulated unhealthy eating habits and the likelihood of obesity and central obesity were stronger in the subgroups of subjects aged 40–59 years, male subjects, and employed subjects than in subjects aged 60–74 years, female subjects, and unemployed subjects (all *p* for heterogeneity < 0.05). In addition, the likelihood of obesity per the number of accumulated unhealthy eating habits was greater in subjects with regular exercise than in those without it. Meanwhile, the association with central obesity tended to be heterogeneous between the drinking habit subgroups (*p* for heterogeneity = 0.09). No clear heterogeneity was detected between the subgroups of smoking habits, marital status, and living status (all *p* for heterogeneity > 0.3).

## 4. Discussion

In the present study, we clearly demonstrated that subjects with unhealthy eating habits—namely, snacking, eating quickly, and eating late-evening meals—had a significantly greater likelihood of the presence of obesity and central obesity. Notably, the accumulation of these unhealthy eating habits was linearly associated with a higher likelihood of obesity and central obesity after adjusting for demographic, socioeconomic, and lifestyle factors in a general Japanese adult population. These findings highlight that unhealthy eating habits, and especially their accumulation, have a major influence on obesity and central obesity, and may suggest that an improvement in these unhealthy eating habits would help to prevent obesity or central obesity.

Epidemiological evidence from cross-sectional and longitudinal studies has shown that individual unhealthy eating habits play a significant role in the development of obesity [5,6,7,8,9,10,11,12]. However, there have been few studies addressing the influence of the accumulation of unhealthy eating habits on obesity or central obesity. Three cross-sectional studies have shown that an accumulation of unhealthy eating habits was positively associated with the prevalence of obesity or metabolic syndrome in adult Japanese populations [12,17,18]. A community-based study conducted in northeast Japan showed that the multivariate-adjusted OR for obesity increased with an increase in the number of unhealthy eating habits, which in this case were skipping breakfast, eating quickly, and eating late-evening meals [18]. These findings were similar to our findings. In addition, one longitudinal study of working adults revealed that individuals who had both of two unhealthy eating habits—namely, snacking after dinner and eating late-evening meals—had an approximately twofold greater likelihood of having obesity than those with only one or neither of these habits [11]. Therefore, it seems reasonable to speculate that the accumulation of multiple unhealthy eating habits might increase the likelihood of obesity and central obesity in Japanese adults.

There are several possible mechanisms underlying the significant association between individual unhealthy eating habits and the greater likelihood of having obesity or central obesity. Subjects with a snacking habit were shown to have a higher total energy intake than those without a snacking habit [19,20]. In general, snacks tend to be high in calories, carbohydrates, and fats [21]. In the US population, the number of individuals with a snacking habit has increased over the last 30 years, and the energy intake from snacking has been estimated to be approximately 280 kcal/day, which is equivalent to 15.4% of the average energy intake of US adults [22,23]. In addition, eating speed is likely to influence the blood concentrations of appetite suppressant hormones. Subjects who eat quickly have been reported to have lower blood concentrations of pancreatic or gut hormones that are expected to suppress appetite (e.g., insulin, glucagon-like peptide 1, and peptide YY) than those who do not [24]. A systematic review revealed that subjects who ate quickly had a higher energy intake than those who ate slowly [25]. These results suggest that subjects with the unhealthy eating habit of eating quickly are more likely to have a higher energy intake due to an increased appetite than those without this habit. Meanwhile, eating late-evening meals has been considered to lead to an energy surplus, because diet-induced thermogenesis is lower at night than in the daytime [26,27]. Moreover, the sympathetic nerve activation and subsequent sleep disturbance caused by increased leptin secretion after a meal may be involved in the excess risk of obesity from eating late-evening meals [28,29]. Insufficient sleep leads to elevated ghrelin, which is an orexigenic peptide that may increase appetite [30]. As we noted above, several independent mechanisms, including behavioral, endocrine, and energy metabolic mechanisms, may be at play in the relation between unhealthy eating habits and obesity, and therefore the accumulation of unhealthy eating habits might additively increase the likelihood of having obesity or central obesity.

In the present study, the subgroup analysis showed that the magnitude of the influence of the accumulation of unhealthy eating habits on the excess likelihood of having obesity or central obesity were stronger in the groups of middle-aged subjects, male subjects, current drinkers, subjects who did not perform regular exercise, and subjects who currently engaged in work than in their counterpart groups (non-middle-aged subjects, female subject, etc.). In general, middle-aged male subjects tend to have a greater energy intake than older subjects and/or women [31,32]. The significant heterogeneity in obesity and central obesity observed between subjects in the current drinking and non-current drinking subgroups and subjects in the employed and unemployed subgroups may also reflect the high energy consumption: Middle-aged men are more likely to have a drinking habit and to be employed. In addition, the absence of a regular exercise habit could contribute to decreased energy expenditure, resulting in a further energy surplus. Our findings suggest that middle-aged male subjects and subjects who do not perform exercise regularly are more likely to be affected by the adverse effects of accumulated unhealthy eating habits on obesity.

The present study has several limitations. First, because of the cross-sectional nature of this study, we were not able to determine whether there was a causal association between unhealthy eating habits and either obesity or central obesity. Second, the information about the unhealthy eating habits was derived from questioning the participants rather than observing their actual behaviors. However, a moderate-to-high level of concordance between the self-reported and friend-reported rate of eating was shown in a previous study [33]. Finally, we did not have information on the energy intake and nutrients. Further studies will be needed to assess these parameters carefully in order to clarify whether unhealthy eating habits increase the likelihood of obesity and central obesity through excessive intake of energy, fat, and carbohydrates.

## 5. Conclusions

The present study revealed dose–response-positive associations between the number of accumulated unhealthy eating habits and the likelihood of obesity and central obesity in a general Japanese population. Our findings suggest that modifying individual unhealthy eating habits and avoiding their accumulation might reduce the burden of obesity and central obesity. Healthcare professionals need to encourage those who have unhealthy eating habits to modify each of their habits individually as well as to avoid accumulating multiple unhealthy habits. Further longitudinal studies will be needed to elucidate whether a causal relationship exists between the accumulation of unhealthy eating habits and the incidence of obesity or central obesity.

## Figures and Tables

**Table 1:** Age- and sex-adjusted participant characteristics according to the status of each unhealthy eating habit.

	Snacking			Eating Quickly			Eating Late-Evening Meals		
	No	Yes	*p* Value	No	Yes	*p* Value	No	Yes	*p* Value
	(*n* = 832)	(*n* = 1074)		(*n* = 1037)	(*n* = 869)		(*n* = 1399)	(*n* = 507)	
Age, year	60.8 (0.3)	60.0 (0.3)	0.11	61.3 (0.3)	59.1 (0.3)	<0.001	61.8 (0.3)	56.4 (0.4)	<0.001
Women, %	40.5	68.3	<0.001	57.8	54.3	0.13	63.1	37.3	<0.001
Current smoking, %	15.9	11.5	0.005	14.3	12.2	0.16	12.2	16.9	0.006
Current drinking, %	64.6	50.0	<0.001	57.6	54.8	0.27	53.2	65.1	<0.001
Regular exercise, %	16.0	15.5	0.77	15.1	16.4	0.42	16.3	14.1	0.27
Married, %	80.2	83.7	0.06	80.9	83.7	0.11	82.7	80.8	0.38
Living alone, %	7.1	4.9	0.04	6.2	5.4	0.44	5.0	8.2	0.02
Current employment, %	52.2	45.9	0.03	47.3	50.2	0.29	44.8	60.0	<0.001

Values are expressed as adjusted mean (standard error), or frequency. Mean values of age were adjusted for sex. Frequencies of women were adjusted for age.

**Table 2:** Multivariable-adjusted likelihood of the presence of obesity and central obesity according to the status of each unhealthy eating habit.

Outcomes Unhealthy Eating Habits	Age- and Sex-Adjusted Mean (95% CI) of BMI or WC	No. of Obese or Central Obese Subjects/Total Subjects	Model 1 ^a)^		Model 2 ^b)^	
			OR (95% CI)	*p* Value	OR (95% CI)	*p* Value
***Obesity***	**BMI (kg/m^2^)**					
**Snacking**						
No	22.8 (22.5–23.0) ^c)^	200/832	1.00 (reference)		1.00 (reference)	
Yes	23.5 (23.2–23.7) ^c),^**	304/1074	1.50 (1.20–1.86)	<0.001	1.49 (1.19–1.86)	<0.001
**Eating quickly**						
No	22.6 (22.3–22.8) ^c)^	207/1037	1.00 (reference)		1.00 (reference)	
Yes	23.9 (23.7–24.1) ^c),^**	297/869	2.12 (1.72–2.61)	<0.001	2.11 (1.71–2.61)	<0.001
**Eating late-evening meals**						
No	23.1 (22.9–23.3) ^c)^	342/1399	1.00 (reference)		1.00 (reference)	
Yes	23.4 (23.1–23.7) ^c)^	162/507	1.38 (1.09–1.74)	0.008	1.39 (1.09–1.77)	0.007
***Central obesity***	**WC (cm)**					
**Snacking**						
No	82.9 (82.2–83.5) ^d)^	315/832	1.00 (reference)		1.00 (reference)	
Yes	84.7 (84.1–85.3) ^d),^**	545/1074	1.30 (1.06–1.58)	0.01	1.29 (1.05–1.58)	0.01
**Eating quickly**						
No	82.4 (81.9–83.0) ^d)^	413/1037	1.00 (reference)		1.00 (reference)	
Yes	85.7 (85.1–86.3) ^d),^**	447/869	1.88 (1.55–2.29)	<0.001	1.89 (1.55–2.30)	<0.001
**Eating late-evening meals**						
No	83.6 (83.1–84.1) ^d)^	645/1399	1.00 (reference)		1.00 (reference)	
Yes	84.7 (83.9–85.6) ^d),^*	215/507	1.35 (1.07–1.70)	0.01	1.36 (1.08–1.72)	0.009

Abbreviations: BMI, body mass index; OR, odds ratio; CI, confidence interval; WC, waist circumference. ^a)^ Adjusted for age and sex. ^b)^ Adjusted for age, sex, current smoking, current drinking, regular exercise, marital status, living status, and employment status. ^c)^ The values are shown as the age- and sex-adjusted mean values (95% CI) of BMI (unit: kg/m^2^). ^d)^ The values are shown as the age- and sex-adjusted mean values (95% CI) of WC (unit: cm). * *p* < 0.05, ** *p* < 0.01 vs. “No”.

**Table 3:** Age- and sex-adjusted characteristics of the study participants according to the number of accumulated unhealthy eating habits.

	Number of Unhealthy Eating Habits				
	0	1	2	3	*p* for Trend
	(*n* = 367)	(*n* = 779)	(*n* = 609)	(*n* = 151)	
Age, year	63.3 (0.5)	61.0 (0.3)	58.7 (0.4)	56.2 (0.8)	<0.001
Women, %	45.6	61.7	57.2	49.5	0.20
Current smoking, %	14.1	14.3	11.5	13.9	0.36
Current drinking, %	60.6	57.2	52.6	56.0	0.06
Regular exercise, %	15.5	16.4	14.8	16.2	0.81
Married, %	76.9	83.0	85.6	76.9	0.12
Living alone, %	6.2	6.3	4.8	7.2	0.64
Current employment, %	46.4	46.7	51.1	54.4	0.08

Values are expressed as adjusted mean (standard error), or frequency. Mean values of age were adjusted for sex. Frequencies of women were adjusted for age.

**Table 4:** Age- and sex-adjusted odds ratios and 95% confidence intervals of the presence of obesity and central obesity per every one increment in the number of accumulated unhealthy eating habits in the subgroups of covariates.

Subgroups	Obesity				Central Obesity			
	No. of Events	No. of Subjects	OR (95% CI) per 1 Increment in the Number of Unhealthy Eating Habits	*p* for Hetero.	No. of Events	No. of Subjects	OR (95% CI) per 1 Increment in the Number of Unhealthy Eating Habits	*p* for Hetero.
**Age**								
40–59 years	186	757	1.98 (1.60–2.45)		298	757	1.71 (1.42–2.05)	
60–74 years	318	1149	1.40 (1.20–1.63)	0.01	562	1149	1.27 (1.10–1.47)	0.01
**Sex**								
Men	267	835	1.75 (1.48–2.08)		243	835	1.63 (1.37–1.93)	
Women	237	1071	1.41 (1.17–1.69)	0.02	617	1071	1.32 (1.13–1.54)	0.007
**Current smoking**								
No	413	1576	1.55 (1.35–1.78)		760	1576	1.44 (1.27–1.64)	
Yes	91	330	1.77 (1.33–2.35)	0.38	100	330	1.53 (1.16–2.01)	0.47
**Current drinking**								
No	214	855	1.55 (1.27–1.88)		439	855	1.31 (1.11–1.56)	
Yes	290	1051	1.62 (1.38–1.91)	0.66	421	1051	1.58 (1.36–1.85)	0.09
**Regular exercise**								
No	422	1595	1.69 (1.47–1.93)		741	1595	1.51 (1.33–1.72)	
Yes	82	309	1.20 (0.90–1.62)	0.046	119	309	1.21 (0.90–1.61)	0.22
**Marital status**								
Unmarried, divorced, and widowed	77	346	1.45 (1.94–1.92)		150	346	1.35 (1.05–1.74)	
Married	427	1560	1.63 (1.42–1.87)	0.49	710	1560	1.48 (1.30–1.69)	0.47
**Living status**								
Living with others	477	1790	1.62 (1.43–1.84)		807	1790	1.47 (1.30–1.65)	
Living alone	27	116	1.23 (0.75–2.03)	0.34	53	116	1.35 (0.85–2.14)	0.66
**Employment status**								
Unemployed	239	983	1.39 (1.16–1.66)		513	983	1.25 (1.07–1.47)	
Employed	265	923	1.82 (1.53–2.17)	0.048	347	923	1.71 (1.45–2.02)	0.006

Abbreviations: OR, odds ratio; CI, confidence intervals; hetero., heterogeneity.
